# Where is the money? The healthcare education and training tariff

**DOI:** 10.1016/j.fhj.2025.100451

**Published:** 2025-07-25

**Authors:** Waqas Akhtar, Alan Roy Abraham

**Affiliations:** a Guy’s & St Thomas’ Foundation NHS Trust, GSTT, London, United Kingdom; b University Hospitals Bristol and Weston NHS Foundation Trust, UHB, Bristol, United Kingdom

**Keywords:** NHS, Health economics, Medical education

## System challenges

The challenges facing the NHS are undeniably immense, particularly in light of the wider economic conditions. Medical education can often seem less of a priority, especially when addressing immediate infrastructure concerns. However, it is crucial to recognise that the future of healthcare hinges on the proper training of our doctors today. The recent NHS England diagnostic ‘Medical training review’^1^ seeks to address the notable gap left by the exclusion of an effective medical training review from the NHS’s 10 Year Plan^2^ and Long Term Workforce Plan.^3^ Unlike the 10 Year Plan, the diagnostic review has no commitment to any resource, and given the current financial outlook it seems unlikely that significant additional funding will be forthcoming.

Therefore, it seems prudent to first focus on ensuring that existing resources are being effectively utilised. However, clarity at national, regional and local levels remains limited, largely because decision-makers do not yet have consistent, accurate information on where and how the money is being spent. In England, the healthcare education and training tariff^4^ is valued at over a billion pounds, with the 2024/25 allocations set at £34,355 per medical student and approximately £13,337, plus basic salary contributions (eg £25,136 on starting core training), for postgraduate doctors in training posts. This funding should be strategically directed to provide both clinical and non-clinical training in the most effective manner possible. Trusts are required to submit yearly accountability reports to regulatory bodies, which are subject to Freedom of Information requests. However, accessing these reports or obtaining clarity on their contents is extremely challenging, and the quality assurance provided remains poor.

## Financial accountability

Where is the money going? When organisations are approached, generic statements are offered such as support for hospital libraries or general facilities. There is even less clarity about funding for medical educators who, many of us know, are giving up their own free time to deliver teaching and training. Too often there are cases of time nominally allocated for educational supervision and training being used to cover gaps and demand in clinical services instead. The medical educator workforce is now the most affected by burnout, based on the latest GMC workforce report.^5^ With lack of sufficient oversight, there is a real concern that a significant portion of this money is being diverted to support NHS trust deficits, as opposed to its intended purpose. This has led to a status quo where the apparent cost of medical training is likely being inflated, as allocated funds are not being used efficiently to deliver education or being diverted for the delivery of services. This lack of quality assurance has created a broken incentive structure surrounding education. There is no hope for system leaders to prioritise where scarce public funding should be allocated when there is no transparency about the substantial current budget.

The integration of the central education and training body Health Education England into NHS England (NHSE) in 2023 introduced constraints in addressing this budget. Just as capital spend in previous years, the education budget in a combined body faced the same sharp end of trade-offs when weighed against acute NHS crises. Even where there was interest in influencing this, NHSE was still limited in its ability to influence at a local level. With the announced dissolution of NHSE, it remains unclear what the future of national oversight for education and training will look like.

## Call to action

It is imperative that all trusts are supported to fulfil their statutory obligation to provide detailed reports on the quality assurance of the healthcare education and training tariff. Directors of medical education should be adequately empowered within the trust’s executive structure, such as a board-level position, to lead these efforts. Effective governance of these funds should actively involve medical students and resident doctors with a robust voice to monitor the allocation of these funds locally, ensuring both transparency and accountability. With the current uncertainty about the future of core education and training roles, there is an even greater urgency in developing improved accountability structures to better embed this at a local and regional level.

We believe that structural reform to support better governance of the healthcare education and training tariff should be a central component of the ongoing national training review and the ongoing restructure of the NHS following the dissolution of NHSE. This should be approached in an open, constructive manner that allows honest discussion about any past mistakes. While we appreciate the difficult financial position and resulting challenging decisions that integrated care boards face today, attempting to resolve the NHS’s current issues without investing in its future workforce is self-defeating. It is up to the medical community to demand answers about where these resources are being directed, quality assure their use in the NHS and ensure that education has a seat at the highest levels of trust governance. We cannot fix the foundations of the NHS without securing the education of its future workforce.

## Funding

This research did not receive any specific grant from funding agencies in the public, commercial or not-for-profit sectors.

## CRediT authorship contribution statement

**Waqas Akhtar:** Writing – original draft, Conceptualization. **Alan Roy Abraham:** Writing – review & editing, Conceptualization.

## Declaration of competing interest

The authors declare the following financial interests/personal relationships which may be considered as potential competing interests: WA was past chair and ARA is currently chair designate of the Academy of Medical Royal Colleges Resident Doctors Committee.
